# Focused ultrasound radiosensitizes human cancer cells by enhancement of DNA damage

**DOI:** 10.1007/s00066-021-01774-5

**Published:** 2021-04-22

**Authors:** Xinrui Zhang, Mariana Bobeica, Michael Unger, Anastasia Bednarz, Bjoern Gerold, Ina Patties, Andreas Melzer, Lisa Landgraf

**Affiliations:** 1 Innovation Center Computer Assisted Surgery (ICCAS), University of Leipzig, Semmelweisstr. 14, Haus 14, Leipzig, 04103 Germany; 2 Institute for Medical Science and Technology (IMSaT), University of Dundee, Wilson House, 1 Wurzburg Loan, Dundee MediPark, Dundee, DD2 1FD UK; 3 Extreme Light Infrastructure - Nuclear Physics ELI-NP, “Horia Hulubei” National Institute for Physics and Nuclear Engineering, 30 Reactorului Street, Bucharest-Magurele, 077125 Romania; 4 Theraclion, 102 Rue Etienne Dolet, Malakoff, 92240 France; 5 Department of Radiation Oncology, University of Leipzig, Stephanstr. 9a, Leipzig, 04103 Germany

**Keywords:** FUS, Hyperthermia, Radiation therapy, Apoptosis, DNA double-strand breaks

## Abstract

**Purpose:**

High-intensity focused ultrasound (HIFU/FUS) has expanded as a noninvasive quantifiable option for hyperthermia (HT). HT in a temperature range of 40–47 °C (thermal dose CEM43 ≥ 25) could work as a sensitizer to radiation therapy (RT). Here, we attempted to understand the tumor radiosensitization effect at the cellular level after a combination treatment of FUS+RT.

**Methods:**

An in vitro FUS system was developed to induce HT at frequencies of 1.147 and 1.467 MHz. Human head and neck cancer (FaDU), glioblastoma (T98G), and prostate cancer (PC-3) cells were exposed to FUS in ultrasound-penetrable 96-well plates followed by single-dose X‑ray irradiation (10 Gy). Radiosensitizing effects of FUS were investigated by cell metabolic activity (WST‑1 assay), apoptosis (annexin V assay, sub-G1 assay), cell cycle phases (propidium iodide staining), and DNA double-strand breaks (γH2A.X assay).

**Results:**

The FUS intensities of 213 (1.147 MHz) and 225 W/cm^2^ (1.467 MHz) induced HT for 30 min at mean temperatures of 45.20 ± 2.29 °C (CEM43 = 436 ± 88) and 45.59 ± 1.65 °C (CEM43 = 447 ± 79), respectively. FUS improves the effect of RT significantly by reducing metabolic activity in T98G cells 48 h (RT: 96.47 ± 8.29%; FUS+RT: 79.38 ± 14.93%; *p* = 0.012) and in PC-3 cells 72 h (54.20 ± 10.85%; 41.01 ± 11.17%; *p* = 0.016) after therapy, but not in FaDu cells. Mechanistically, FUS+RT leads to increased apoptosis and enhancement of DNA double-strand breaks compared to RT alone in T98G and PC-3 cells.

**Conclusion:**

Our in vitro findings demonstrate that FUS has good potential to sensitize glioblastoma and prostate cancer cells to RT by mainly enhancing DNA damage.

**Supplementary Information:**

The online version of this article (10.1007/s00066-021-01774-5) contains supplementary material, which is available to authorized users.

## Introduction

High-intensity focused ultrasound (HIFU) or focused ultrasound (FUS) plays an increasing role in medical applications because of thermal and mechanical effects on cells, biological molecules, and tissue, thus expanding the traditional application of ultrasound in diagnostic imaging to therapeutic applications [1, 2]. Various magnetic resonance imaging (MRI)- and ultrasound imaging (US)-guided HIFU systems are CE marked and approved by the FDA (Food and Drug Administration) for treatment of benign uterus myomas, ablation of prostate tissue, palliative pain management of bone metastasis, and neurological indications like essential tremor [3, 4]. While hyperthermia (HT) and tissue ablation with HIFU/FUS are technically feasible therapeutic methods [5], there is great potential with low FUS intensity less than 5 W/cm^2^ for FUS-mediated delivery of therapeutic agents [6–9] including chemotherapeutics, genetic material [10], proteins, and small molecules to cells and solid tumors [11]. FUS can deliver various levels of acoustic energy at desired sites in the body with minimal damage to tissue adjacent to the target. As mentioned above, MRI-guided HIFU/FUS enables not only the generation of high temperatures (> 55 °C) to induce coagulation and tissue necrosis, but it also provides a method to generate quantifiable and local HT (40–47 °C) in the target area [12].

In oncology, surgery, image-guided ablation, local radiation therapy (RT), and systemic chemotherapeutics are the four clinical treatment modalities, but all are accompanied by significant adverse effects [13, 14]. RT commonly used in the management of head and neck tumors causes skin burns and affects voice, jawbone, and teeth [15]. Glioblastoma is highly resistant to clinical radio- and chemotherapy and is often inoperable, leading to low survival rates [16, 17]. Thermal ablation is usually not applicable either. Prostate cancer is the most common cancer in males in Europe and metastatic prostate cancer can lead to incapacitating pain and fractures. As examples of highly aggressive cancer entities, head and neck tumors, glioblastoma, and metastatic prostate cancer subtypes require novel and more effective treatment regimens, and were thereby chosen in our study.

To enhance the treatment outcome, reduce radiation dose, and simultaneously avoid radiation-induced adverse effects, growing interest lies on the radiosensitization of the tumor cells. Heating of tumor cells up to high temperatures (40–47 °C) in a thermal dose cumulative equivalent minutes at 43 °C (CEM43) range of 25–200 min showed beneficial radiosensitizing effects [12, 18]. The underlying mechanism of RT is the induction of DNA double-strand breaks leading to cell death [19]. Application of HT was reported to increase the tumor radiosensitivity by enhancing DNA damage, inhibition of DNA repair mechanism, and reduction of hypoxia [20, 21]. Different mechanisms like induction of apoptosis mediated by an inhibition of heat shock proteins [22, 23] or directly by damage of DNA [24] are also described as leading to radiosensitization effects.

Currently, HT is mostly delivered by microwave, radiofrequency, or FUS in clinic. Microwave and radiofrequency generate heating by electromagnetic waves causing rotation of water molecules in cells, inducing frictional heating [25]. In early 1989, the effect of combined water bath HT and RT was published: higher cytotoxicity was observed 24 h after exposure to 43 °C for 1 h and single-dose radiation at 5–10 Gy compared to RT alone [26]. Moreover, irradiation of 10 Gy and microwave-induced HT at 44 °C for 1 h were reported to significantly enhance cell death of breast cancer cells 72 h after treatment [27]. The described effects of HT are intensively studied experimentally in vitro and in vivo in a high variety of cancer models [21]. Nevertheless, the electromagnetic wave-based HT methods lack reproducibility in target precision and heating capacity, and do not allow the quantification of the thermal dose through MR temperature mapping. Therefore, electromagnetic wave-based HT is not accepted as a standard procedure in clinical practice. Main challenges to establishing HT are the invasiveness of temperature control techniques, generation of uncontrollable heat spots, and focused heating with adequate penetration depth. Electromagnetic waves cannot be focused as precisely as is possible with ultrasound waves.

In contrast, ultrasound waves are mechanical waves generating heat through mechanical friction, which has the advantage of tissue penetration up to 20 cm. When FUS propagates through tissue or cells, the acoustic intensity decreases as the energy of the wave is absorbed by the medium and as a result, local heating occurs. FUS is the only noninvasive technique in the thermal therapy field [28]. Besides, the main advantage of FUS-induced HT is the precise treatment planning under MRI guidance, which allows limited heating in the target region without damage to surrounding tissues. However, due to the lack of a reproducible in vitro FUS system, only a few studies have reported combined FUS and RT, and the biological effects and mechanisms are not sufficiently investigated.

To translate the benefits of FUS-induced HT to support RT, a certain number of experiments in vitro are required to form the basis for preclinical in vivo studies. However, studies regarding the interaction of FUS fields with cells and comparative evaluation is difficult due to the lack of a standardized technique for FUS delivery. On the other hand, the attenuation and absorption of acoustic energy are much less in cell culture medium compared to soft tissues. For instance, cancer cells were exposed to ultrasound in centrifuge tubes in an in vitro study using a clinical HIFU system [29]. The complicated handling of the clinical system makes the experiment with various ultrasound parameters difficult. We developed a high-throughput laboratory in vitro FUS system that mimics the principles of clinical application, allowing the generation of FUS-induced HT in a standard-size 96-well plate. Using the device can reduce instrument-related variability in the results, improve the reproducibility of acoustic fields, and facilitate the translation of results to in vivo experiments and, ultimately, to medical practices.

This study aimed to investigate the short-term radiosensitizing properties of FUS-induced HT at the molecular level with a newly developed in vitro experimental setup. The impact of combination treatment including FUS and X‑ray irradiation on human glioblastoma, head and neck tumor, and prostate cancer on cellular metabolic activity, induction of apoptosis, DNA double-strand breaks, and cell cycle phase changes was investigated.

## Methods

### Tumor cell lines and cell culturing

Human glioblastoma cell line T98G was purchased from American Type Culture Collection (ATCC) and LN405 from German Collection of Microorganisms and Cell Culture GmbH (DSMZ). Cells were cultured in Dulbecco’s Modified Eagle’s Medium (DMEM, Gibco, Thermo Fisher Scientific, Darmstadt, Germany). Human prostate cancer cell line PC‑3 was purchased from the European Collection of Authenticated Cell Cultures (ECACC, Salisbury, UK) and grown in Ham’s F‑12K (Kaighn’s) medium (Gibco, Thermo Fisher Scientific, Darmstadt, Germany). The head and neck cancer cell line FaDu and UTSCC‑8 (OncoRay, National Center for Radiation Research in Oncology, Dresden, Germany) were cultured in DMEM medium supplemented with 2% HEPES (1 M, PAA Laboratories, Pasching, Austria), 1% sodium pyruvate (100 mM, Sigma-Aldrich GmbH, Munich, Germany), 1% MEM non-essential amino acids (100 ×, Sigma-Aldrich GmbH, Munich, Germany). All cell culture media were supplemented with 10% (v/v) fetal bovine serum (FBS, Gibco, Thermo Fisher Scientific, Darmstadt, Germany), 100 U/mL penicillin, and 100 mg/mL streptomycin (Biochrom GmbH, Berlin, Germany) for culturing of the cell lines at 37 °C in a humidified incubator supplemented with 5% (v/v) CO_2_. For experiments, cells were routinely washed with phosphate-buffered saline (PBS, Biozym Scientific GmbH, Hessisch Oldendorf, Germany), and detached using trypsin/EDTA (Biozym Scientific GmbH, Hessisch Oldendorf, Germany). Cells used in this experiment were in the exponential growth phase and the passage number was less than 20. The cells were negative for mycoplasma as routinely determined via PCR.

### Focused ultrasound in vitro setup

**Fig. 1:**
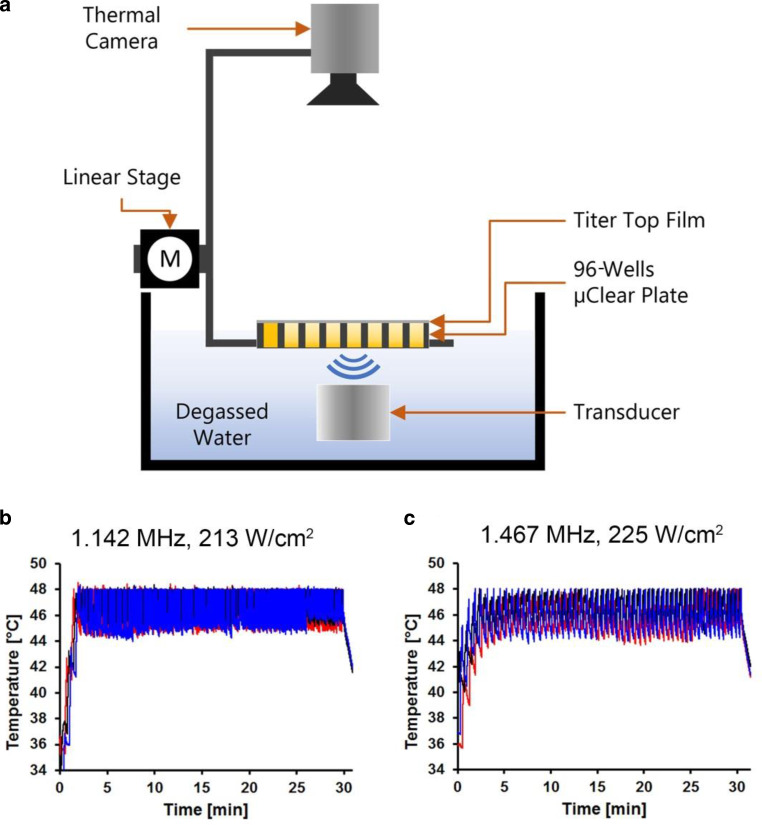
The in vitro focused ultrasound (FUS) system. **a** For FUS exposure the ultrasound signal was generated by a piezoelectric focused transducer immersed at the bottom of a tank filled with degassed water. The transducer is driven by a signal generator (Agilent 33120A) and an RF power amplifier (Electronics and Innovation A075). Alongside the water bath, an X‑Slide linear stage connected to a programmable motor controller and a stepper motor (VELMEX NEMA 17) translates an attached Perspex holder carrying a cell culture well plate. The whole assembly slides through the water above the transducer on two parallel aluminum tracks using small linear bearing carriages (Drylin N). A thermal camera monitors temperature in real time inside the wells with a feedback loop to the linear stage to enable FUS in 3 wells in parallel. **b** The temperature generated by the FUS transducer with frequency of 1.142 MHz at intensity of 213 W/cm^2^ to the mean temperature of 45.20 ± 2.29 °C and **c** for frequency of 1.467 MHz at intensity of 225 W/cm^2^ to the mean temperature of 45.59 ± 1.65 °C was measured by infrared thermal camera. The *blue*, *black*, and *red lines* represented the real-time temperatures in three in-parallel sonicated wells

The FUS in vitro system (Fig. 1a) consists of a Perspex box with separate compartments for water where the ultrasound transducer and the cell culture 96-well µClear plates are placed. Deionized water was boiled for 5 min and cooled down to room temperature in a sealed container for degassing [30]. Degassed water was circulated in the water tank with a self-priming water pump (Lei Te Co., Ltd., Guangdong, China) to reduce ultrasound artifacts and prevent air bubble formation underneath the plate. Temperature was kept close to physiological levels at 34 °C using an external heater (ETH 200, Hydor UK, Shropshire, UK). The temperature was maintained 3 °C lower than human body temperature to avoid neighbor overheating and formation of air bubbles. To reach the target temperature quickly, two transducers with frequencies of 1.142 and 1.467 MHz at acoustic intensities of 213 and 225 W/cm^2^ were chosen in this study to reach the target temperature of 45 °C (Fig. 1b, c). These two transducers are geometrically focused with piezoceramic bowls (Meggitt-Ferroperm Piezoceramics, Kvistgaard, Denmark). The focus points of the transducers fit into the size of one well of the 96-well plate, 6.96 mm in diameter and 10.9 mm in depth, when the focus point is positioned at the bottom of the well (Supplementary Table S1, Fig. S1). The acoustic intensities at 1.14 and 1.467 MHz inside the well were calibrated with hydrophone (Supplementary Table S2 and S3). The real-time temperature in the wells during FUS treatment was monitored using an infrared thermal camera PI450 (Optris GmbH, Berlin, Germany) and imaging software (PI connect version 2.10) with a feedback loop to the motor (VELMEX Inc., Bloomfield, NY, USA) for the treatment of three non-adjacent wells simultaneously.

As the thermal dose plays a major role in most hyperthermia research [31], the cumulative number of equivalent minutes at 43 °C (CEM43) in terms of the thermal dose was used to quantify thermal exposure during treatment (Table 1). It was calculated according to the equation published by A. Szasz et al. [32].

1 $$\begin{array}{c} \mathrm{CEM}43=\int _{0}^{t}R^{[43-T\left(r,\tau \right)]}d\tau \\ R=\left\{\begin{array}{c} 0,if\:T<39^\circ C\\ 0.25,if39\leq T<43^\circ C\\ 0.5,ifT\geq 43^\circ C \end{array}\right. \end{array}$$

**Table 1:** Sonication duration per well and thermal dose

Frequency [Hz]	Mean temperature [°C]	Sonication duration per well [min]	Thermal dose (CEM43)
1.142	45.20 ± 2.29	10.06 ± 0.80	436 ± 88
1.467	45.59 ± 1.65	10.18 ± 0.31	447 ± 79

### Experimental protocol for in vitro focused ultrasound treatment

Cells were cultured as described above and seeded into special ultrasound-penetrable 96-well cell culture plates with a 200 µm thin foil µClear bottom (Greiner Bio-One Ltd, Stonehouse, UK). FaDu and T98G cell lines at cell density in a range of 2500–5000 cells/well, and PC-3 cells at cell density in a range of 5000–10,000 cells/well were seeded in the corresponding cell culture medium to reach 90–100% confluency at the desired timepoint after treatment. The seeding was performed 1 day prior to the FUS treatment. Before sonication, up to 420 µL/well of cell culture medium was added to each well and the plate was sealed with Titer-tops (Electron Microscopy Sciences, Hatfield, PA, USA) plate-sized US transparent films, avoiding air bubble formation. The plate was placed in the FUS system using the plate holder located above the transducer. FUS was applied continuously for 30 min at intensities of 213 W/cm^2^ (1.147 MHz) or 225 W/cm^2^ (1.467 MHz) to hold the temperature stable at mean temperature of 45.20 ± 2.29 °C (1.147 MHz) and 45.59 ± 1.65 °C (1.467 MHz; Fig. 1b, c) for the FUS treatment. A nearly parallel sonication regime was defined in MATLAB® based on the feedback from the camera as follows: (1) move to the well with the lowest temperature; (2) sonicate the well until its temperature reaches the target temperature; (3) repeat until the length of 30 min is reached. After sonication, the cells were observed under optical microscopy, the entire volume of cell culture medium was removed from the plate and refilled with 100 µL/well fresh medium followed by incubation of cells for a further 1–72 h to investigate the short-term effects.

### X-ray irradiation of cancer cell lines

Cells cultured in 96-well plates with corresponding cell density in a range of 2500–10,000 cells/well were irradiated with a single dose using a 150 kV X‑ray machine (DARPAC 150-MC, Raytec Inc., Swindon, UK) at a dose rate of 1.276 Gy/min. For determination of the non-lethal radiation dose, radiation dose curves with 0 to 20 Gy were obtained. To quantify the cell proliferation after irradiation, BrdU assay (Roche, Basel, Switzerland) was performed 96 h post treatment. Based on the preliminary radiation dose curves, a single dose of 10 Gy was used in all combination experiments (Supplementary Fig. S3).

### Treatment protocol of combination of FUS and RT

**Fig. 2:**
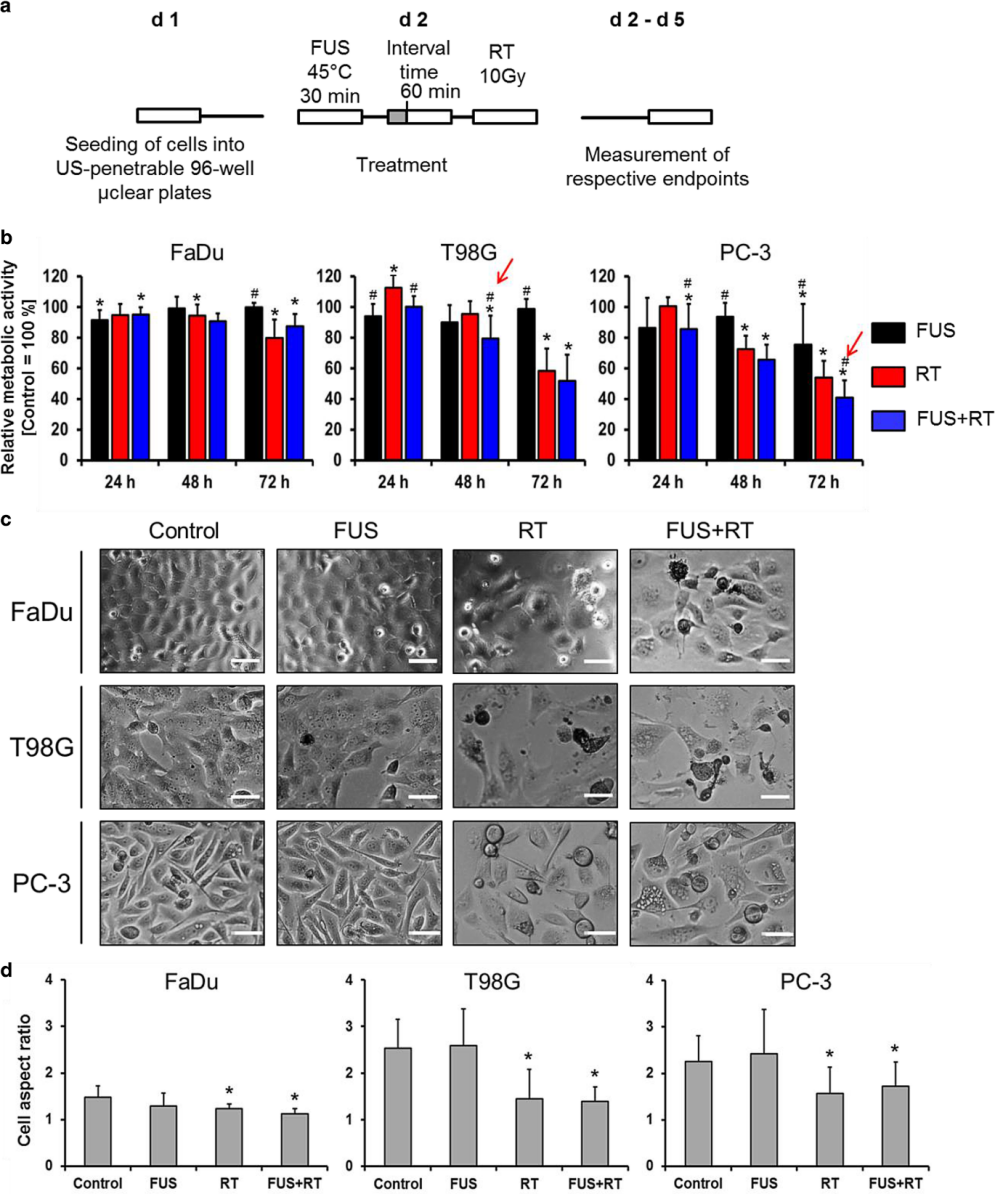
The combination of FUS and RT (10 Gy) decreases the cell metabolic activity in a time-dependent manner. Cancer cell lines were treated with the FUS in vitro system at mean temperature of 45 °C for 30 min followed by irradiation of 10 Gy 60 min later in the combination groups. **a** The experimental timing scale describes the treatment procedure in the FUS and RT combination group. **b** Relative cellular metabolic activity was measured by WST‑1 assay. Data were normalized to the untreated control, which was set as 100%. **c** Representative microscopy images showing alterations in cell morphology 72 h after treatment. Scale bar = 50 µm. **d** Quantification of observed cell aspect ratio 72 h after treatment. Data are presented as mean ± SD. *N* = 9, significantly different from untreated control (*p ≤ 0.05), significantly different from RT (#*p* ≤ 0.05). *FUS* focused ultrasound, *RT* radiation therapy

The cancer cells were seeded in 96-well plates with µClear bottom 24 h before treatment at the density of 2500–10,000 cells/well in the corresponding culture medium. The cells were first exposed to FUS-induced hyperthermia as the protocol described above, followed by single-dose X‑ray irradiation at 10 Gy with an interval time of 60 min (Fig. 2a). After treatment at respective endpoints, cell metabolic activity, apoptosis, cell cycle distribution, and DNA damage were evaluated.

### Microscopic analysis and measurement of metabolic activity

Cell morphology was analyzed by microscopy before and after treatment (ZEISS Axio Observer, Carl Zeiss microscopy GmbH, Jena, Germany). To determine the short-term impact of the different treatments on the metabolic activity of the human cancer cell lines, the tetrazolium salt-based metabolic activity assay WST‑1 (Roche, Basel, Switzerland) was performed 24, 48, and 72 h after treatment. According to the manufactures’ instructions, the medium was discarded and cells were incubated with fresh cell culture medium including WST‑1 reagent (final 10%) in the 96-well µClear cell culture plates. The absorbance at 435 nm was measured using a plate reader (Synergy H1, BioTek, Bad Friedrichshall, Germany).

### Annexin V-FITC assay

To detect apoptotic and necrotic cells an annexin V-FITC assay (Cayman Chemical, Ann Arbor, MI, USA) was performed 4, 24, and 72 h post treatment according to the manufacturers’ instructions. In brief, the staining solution was prepared freshly by mixing annexin V-FITC (1:500) and propidium iodide (PI, 1:2000) with binding buffer. 50 µL staining solution was added to each well and incubated at room temperature for 10 min in the dark, centrifugation for 5 min at 400 g was performed afterwards. Finally, staining solution was discarded and 100 µL binding buffer was added to each well followed by fluorescence microscopy using ZEN 2.3 software (ZEISS Axio Observer, Carl Zeiss microscopy GmbH, Jena, Germany). Apoptotic cells stained with annexin V-FITC were detected at excitation/emission maxima at 485/535 nm and PI-stained dead cells at excitation/emission maxima at 535/617 nm. Flow cytometry (AttuneNxT, Thermo Fisher Scientific, Darmstadt, Germany) was used additionally to quantify the number of stained cells.

### Cell cycle analysis and apoptosis detection

The effects of FUS and RT on apoptosis-induced DNA fragmentation and cell cycle phase distribution were investigated by Nicoletti assay [33] using flow cytometry 4, 24, and 72 h after treatment. Briefly, cells were trypsinized from 96-well plates with 100 µL trypsin/EDTA per well and harvested into one 1.5 ml reaction tube, washed twice with PBS, and fixed with 70% ethanol at −20 °C overnight. Cells were washed again twice with PBS and incubated with 60 µL RNaseA solution (Sigma-Aldrich GmbH, Munich, Germany) at a final concentration of 0.1 mg/mL at 37 °C for 20 min. Afterwards, the DNA content was stained by propidium iodide (PI, Sigma-Aldrich GmbH, Munich, Germany) at a final concentration of 50 µg/mL at 4 °C for 5 min and cells were assessed by flow cytometry (AttuneNxT, Thermo Fisher Scientific, Darmstadt, Germany).

### Detection of DNA double-strand breaks

To determine DNA double-strand breaks (DSBs), γH2A.X assay was performed 1 and 24 h post treatment. The cell culture medium was aspirated from each well, and cells were fixed with 4% formaldehyde at 37 °C for 10 min, chilled on ice for 1 min, the fixative was removed, and cells were washed three times with 1 × PBS. Cells were permeabilized with 90% methanol on ice for 30 min, and again washed three times with PBS. Non-specific antibody binding was blocked by 0.5% bovine serum albumin (BSA, Cell Signalling Technology, Danvers, MA, USA, 100 µL/well) in PBS at room temperature for 10 min. After removing the block solution (0.5% BSA in PBS), cells were incubated with 50 µL/well phospho-histone H2A.X (Ser139) rabbit primary monoclonal antibody (#9718, Cell Signalling Technology, Danvers, MA, USA) at a concentration of 1:400 diluted with block solution at room temperature for 1 h. Cells were washed three times with antibody-free BSA solution and incubated with 50 µL/well secondary antibody (anti-rabbit IgG conjugated with Alexa Fluor® 594 fluorescent dye; #8889, Cell Signalling Technology, Danvers, MA, USA) at a concentration of 1:1000 diluted with block solution at room temperature in the dark for 30 min. Cells were finally washed three times with block solution and mounted with Fluoromount‑G^TM^ (Thermo Fisher Scientific, Darmstadt, Germany) including DAPI to stain cell nuclei. DNA double-strand breaks were visualized at excitation/emission at 561/594 nm and blue nuclei at 358/461 using fluorescence microscopy. The mean numbers of stained foci per cell nucleus were semi-quantitatively analyzed by ImageJ software Find Maxima tool, counting all nuclei (80–120 nuclei) in the images.

### Statistical analysis

Statistical analysis was performed using the statistical program Origin (Origin 6.0, OriginLab, Northampton, Massachusetts, USA). All data of metabolic activity (WST‑1 assay), cell cycle (PI), and DNA double-strand breaks (γH2A.X) are expressed as means ± standard deviation of three independent experiments with three replicates. The significance of difference between two mean values was assessed by one-way ANOVA test. A *p*-value ≤ 0.05 was considered to be statistically significant.

## Results

### Effective combination of FUS and RT leads to reduction in metabolic activity and morphologic changes in glioblastoma and prostate cancer cells

To evaluate the short-term effects on cancer cell morphology and metabolic activity after combination of FUS and RT treatments, cell morphology and cellular metabolic activity were evaluated 24, 48, and 72 h post treatment. The combination of FUS and RT did not affect cell metabolic activity of T98G cells significantly at 24 h, whereas a significant decrease was observed in FaDu (95.28 ± 4.74%) and PC‑3 (85.82 ± 16.44%) cells compared to untreated controls. A significant reduction (*p* ≤ 0.05) of metabolic activity compared to RT alone was observed in T98G cells from 96.47 ± 8.29% (RT only) to 79.38 ± 14.93% (FUS+RT; 48 h) and in PC-3 cells from 54.20 ± 10.85% (RT only) to 41.01 ± 11.17% (FUS+RT; 72 h; Fig. 2b, red arrow). In contrast, no significant changes of metabolic activity in FaDu cells were noticed at 48 and 72 h in the FUS+RT group compared to RT alone. FUS alone only showed a notable effect on metabolic activity in FaDu cells (91.45 ± 6.55%) at 24 h and in PC-3 cells (75.49 ± 26.61%) at 72 h compared to untreated controls, the effect was not observed in T98G cells. A typical cobblestone-like phenotype was observed in all cell lines after ionizing radiation (Fig. 2c). As demonstrated by quantification of morphology, the cell aspect ratio was significantly reduced in the RT-only and FUS+RT group 72 h after treatment compared to the control group (Fig. 2d).

### Combination of FUS and RT triggers programmed cell death

**Fig. 3:**
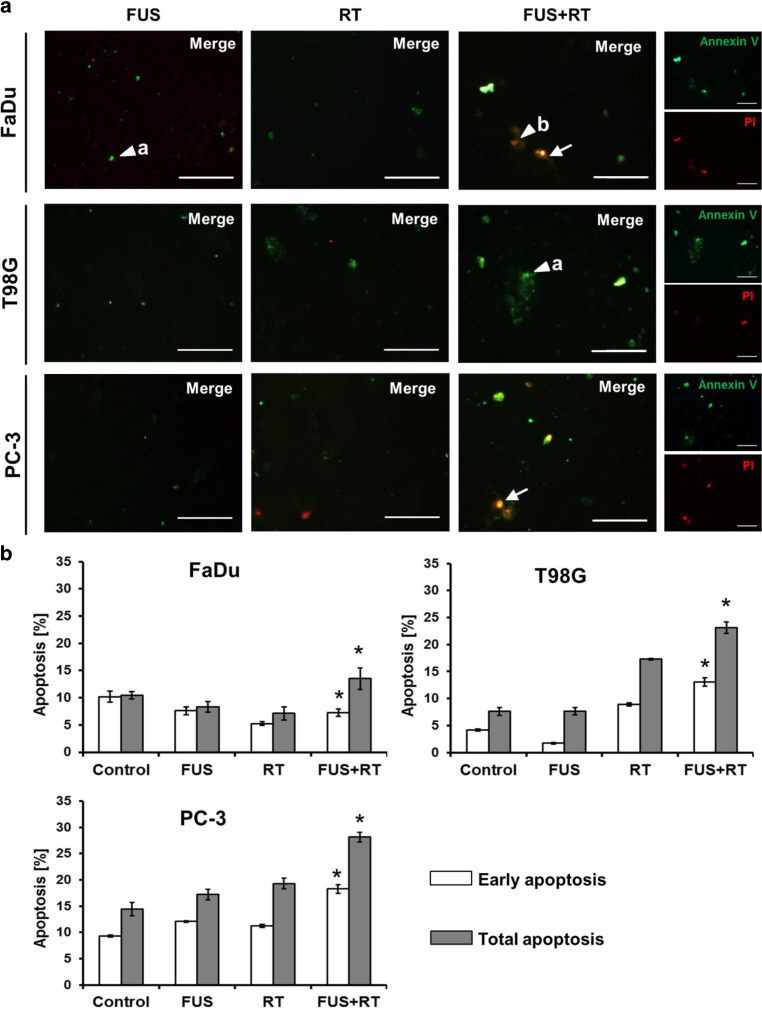
Combination of FUS and RT (10 Gy) enhances apoptotic rate in PC-3 cells. **a** Representative fluorescence microscopy images showing single annexin V-FITC-stained (*green*) early apoptotic cells (*triangle* **a**) and single PI-stained (*red*) necrotic cells (*triangle* **b**). Double staining indicates late apoptotic cells (*white arrows*). Scale bar = 100 µm. **b** Quantified flow cytometric analysis of annexin V‑FITC/PI was shown 72 h post treatment, early apoptosis and total apoptosis are given as percentage of total cells (*p ≤ 0.05, significantly different from RT). *FUS* focused ultrasound, *RT* radiation therapy

Next, we explored whether thermal effects induced with FUS and the combination of FUS and RT are able to cause apoptosis detected via annexin V‑FITC/PI. Representative fluorescence microscopy images revealed no apoptotic or necrotic events in the untreated control (Fig. 3a). FUS alone and RT alone induced only a small number of early (green, annexin V +/PI −) and late apoptotic cells (green and red, annexin V +/PI +). A higher number of late apoptotic cells (annexin V+/PI+) was noticed after combination of FUS and RT compared to RT alone 72 h post combination treatment, especially in T98G and PC-3 cells. Only a few or no necrotic cells (annexin V −/PI +) were observed in FaDu, T98G, and PC-3 cells at this timepoint. To quantify apoptotic rates after treatment, the annexin V-FITC/PI staining was analyzed by flow cytometry in a single experiment 72 h after treatment. The combined FUS and RT treatment (Fig. 3b) showed early apoptotic levels higher by 1.4-fold in FaDu cells, by 1.5-fold in T98G, and by 1.6-fold in PC‑3 compared to RT alone.

### Combination of FUS and RT leads to an increase in apoptosis and G2 phase arrest

To determine the potential role of FUS as a radiosensitizer, the cell cycle phase distribution of cancer cells and the apoptotic sub-G1 fraction indicating apoptotic cells was measured 4, 24, and 72 h after treatment. Compared to the untreated control and FUS alone, the sub-G1 phase cell population was significantly enhanced after RT alone and further enhanced after combined FUS+RT in all three cell lines 72 h post treatment (Table S4). In comparison to untreated controls, a dramatic increase of the sub-G1 population was observed after combined FUS+RT, by 6.3-fold in FaDu after 72 h, 3.8-fold in T98G after 24 h, and 4.4-fold in PC‑3 after 72 h. Compared to RT alone, the sub-G1 fraction was increased by 1.3-fold, 1.8-fold, and 1.6-fold, respectively.

**Fig. 4:**
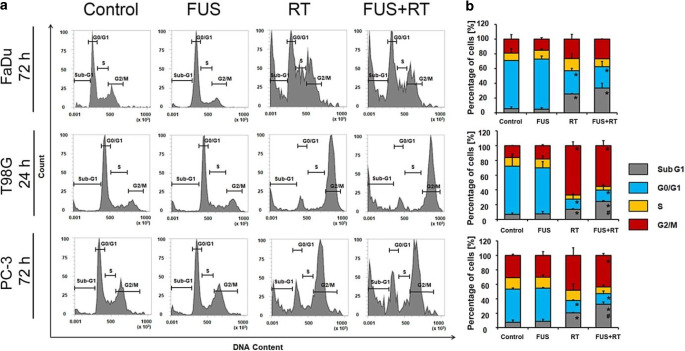
FUS and RT (10 Gy) combination impacts sub-G1 and G2/M cell cycle phase. **a** Representative flow cytometry cell cycle measurements 24 or 72 h post treatment. Depending on different doubling times of the cell lines, different timepoints after treatment are shown in the diagrams. **b** Graphical representation of the cell cycle distribution of FaDu, T98G, and PC-3 cells under control conditions and in response to treatment 24 or 72 h post treatment. Cell populations in sub-G1, G0/G1, S, and G2/M phases are given as percentage of total cells (*p ≤ 0.05, significantly different from control; #*p* ≤ 0.05, significantly different from RT). *FUS* focused ultrasound, *RT* radiation therapy

Changes in cell cycle phase distribution were observed, showing an increased sub-G1 population with a subsequent reduction of G0/G1 cells by approximately 30% in all cell lines after RT and FUS+RT compared to the untreated control. FUS alone showed no significant impact on cell cycle distribution at different timepoints for any cell lines. No regulation of S phase was seen in any single or combination treatment group. The percentage of cells in G2/M phase was enhanced 1.4-fold in FaDu, 3.6-fold in T98G, and 1.4-fold in PC‑3 after FUS+RT treatment compared to untreated controls (Fig. 4b). Interestingly, the populations of T98G and FaDu cells in G2 phase decreased dramatically at 72 h compared to 24 h after RT; however, this reduction was not observed in PC-3 cells (Supplementary Table S2).

### Increase of DNA double-strand breaks after combined FUS and RT

**Fig. 5:**
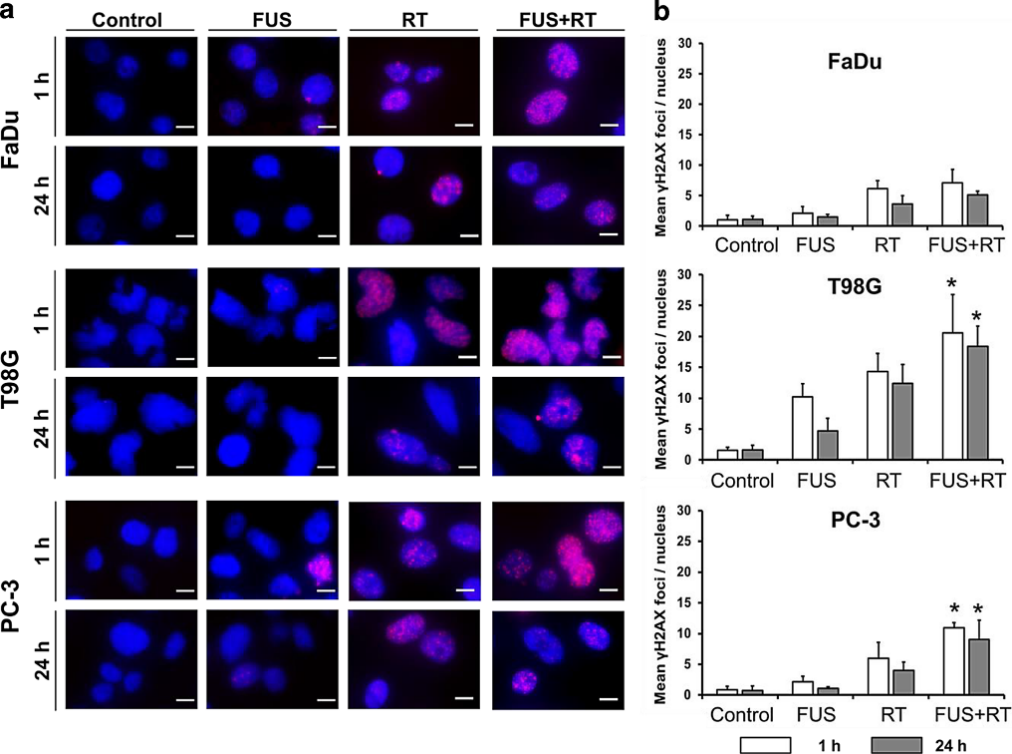
Combination of FUS and RT (10 Gy) enhanced the number of DNA double-strand breaks compared to single treatment. **a** Representative microscopy images showed more γH2A.X foci (Alexaflour568 *red*) in the cell nucleus (*blue*) after combination treatment (FUS+RT) compared to single FUS and single RT treatments. Scale bar = 10 µm. **b** Quantification of γH2A.X foci 1 h and 24 h after treatment. The data represent the mean ± SD, *n* ≥ 6, *significantly different from RT (*p* ≤ 0.05). *FUS* focused ultrasound, *RT* radiation therapy

DSBs were employed to explore the potential of FUS to affect DNA repair mechanisms by scoring the γH2A.X foci 1 and 24 h post treatment (Fig. 5, Table S5). Representative fluorescence microscopy images of stained γH2A.X foci showed the highest number of stained initial (1 h) and residual (24 h) foci in all cell lines in the combination group (FUS+RT) (Fig. 5a, red foci). All cell lines revealed only a limited number of DSBs in the untreated control at low levels ranging from 0.75 to 1.6 foci/nucleus (Fig. 5b). Similar numbers of initial γH2A.X foci per cell were detected after FUS alone in FaDu cells (2.12 foci/nucleus) and PC-3 cells (2.19 foci/nucleus). In contrast, a higher initial γH2A.X number was observed in T98G cells (10.21 foci/nucleus) in the FUS-only treatment group. In comparison to RT alone, significantly higher numbers of initial foci were measured in T98G (1.4-fold) and PC‑3 (1.8-fold) cells after the combination of FUS and RT. The residual foci were scored 24 h post treatment, and displayed similar results with a slight reduction compared to initial foci number. Neither initial nor residual foci enhancement was observed in FaDu cells after FUS+RT treatment compared to RT alone.

## Discussion

Regarding the translation of the combined FUS and RT effect found in this in vitro study to the clinical situation, we first want to mention that the applied frequency range of 1.0 to 1.5 MHz is reasonable, since most of the clinically approved HIFU and FUS platforms provide frequencies of 0.8–1.5 MHz for body applications including, e.g., ablation of uterine fibroids and bone metastasis [3, 34]. Moreover, the clinically available HIFU-HT platform Sonalleve (Profound medical, Canada) works in the described frequency range [35]. Lower frequencies ≤ 0.5 MHz which are, e.g., applied by Exablate (Insightec, Israel) for blood–brain barrier opening [36] or higher frequencies like 3 MHz used in the Echopulse system (Theraclion, France) have only a limited penetration depth [37] and would thus not be suitable for flexible applications in clinical use cases.

Use of a normal polystyrene 96-well plate is not advisable for studying the effects of FUS in vitro, because the FUS beams cannot penetrate but are even absorbed and reflected by the plastic parts. Therefore, special cell culture plates should be used which have a sufficiently thin foil bottom for transfer of the FUS waves to the cells.

The response of cancer cells to combination treatment with HT and RT is dependent on time interval and treatment sequence [38]. As reported in a clinical study, a short time interval (≤ 79.2 min) displayed significantly higher 5‑year survival compared to the long time interval group [39]. With a short interval time of 1 h, the beneficial effects were similar in cancer cells regardless of the treatment sequence [40]. Due to the practical transportation, the FUS treatment was performed before RT with an interval time of 60 min in our study.

Since HT-induced cytotoxicity and DNA damage can be observed during 24 h and these effects can be recovered by time as reported in the literature [41, 42], it is essential to evaluate the cell metabolic activity with same time scale. In contrast, clonogenic assay, which is typically used in radiation research, measures the reproductive cell death by assessing the long-term effect after 2–3 weeks [43]. Therefore, cell metabolic activity was measured within 72 h for investigation of short-term biological effects.

Interestingly, no significant changes were detected on cellular metabolic activity and morphology after the FUS alone treatment. That reveals that FUS at temperatures in a range of 43–47 °C has no cytotoxic effects, indicating that FUS waves do not lead to structural damage or detachment of monolayer cells, even if the thermal dose CEM43 is over 400. Brüningk et al. [44] reported that high-temperature HT induced by PCR thermal cycler at 47 °C (CEM43 up to 700) reduced cell viability by approximately 40% in CAL27 cells (squamous cell carcinoma cell line) if the thermal dose was above 350. FUS treatment induces the thermal effects by mechanical waves, which is different to the PCR thermal cycler and may consequently result in different biological effects. The temperature curve of a PCR thermal cycler is more stable compared to the FUS treatment in our study, which shows rapid temperature fluctuations. During FUS treatment, the temperature changed very fast due to the movement of 96-well plate; therefore, the cells cannot be exposed to the maximum temperature of 48 °C for a long duration, but the thermal dose was dramatically increased in our case. In addition, cells treated by PCR thermal cycler were often in suspension; however, cancer cells are more sensitive to heat treatment in suspension compared to monolayer [45]. Further investigations of FUS regarding different heat induction mechanisms and heating profiles are required in comparison to conventional HT.

The typical cobblestone-like shape of cells was induced mainly by ionizing radiation [46]; the combination of FUS and RT did not lead to significant morphologic changes in the cell aspect ratio compared to RT alone. The significant reduction of cell metabolic activity post FUS+RT treatment in comparison to the RT-only group indicates radio-additive effects of FUS for the investigated glioblastoma and prostate cancer cell lines. This finding is in accordance with a study of McDonald et al., who previously reported about an additive effect on glioblastoma cells when modulated electro-HT was combined with X‑ray radiation [47]. No reduction of metabolic activity was seen in the head and neck cancer cell line FaDu, displaying an overall lower sensitivity of this tumor cell line to combined FUS and RT treatment. In this context, a relatively high resistance of head and neck tumors to radiotherapy is reported in the literature [48]. In the clinic, this circumstance is still a major cause of poor survival rates after RT of head and neck tumors, revealing the need for radiosensitization of this tumor entity.

After HIFU ablation treatment, the tissues are typically in a necrotic state and need to be cleared by the body, which may cause inflammation [49]. Therefore, programmed cell death (apoptosis) avoiding tissue coagulation and necrotic events is preferred using temperatures below 50 °C. Notably, higher numbers of early and late apoptotic cells and the sub-G1 phase cell population were observed 72 h post combination treatment in T98G and PC-3 cells compared to RT alone. The higher number of apoptotic cells after FUS+RT indicates the additive effects of FUS to support RT. In addition, no necrotic cells were noticed, illustrating that necrosis is a rare event after treatment at 72 h. Moreover, fragmentation of DNA is described as a hallmark of apoptosis [50] and was visualized by the sub-G1 peak in the histograms, confirming the results of the annexin V assay. In general, HT has been reported in numerous studies to induce programmed cell death [51], thus enhancing radiosensitivity. Nevertheless, it is possible in our study that the temperature fluctuations in the 96-well plates during the FUS in vitro treatment with the current setup led to only a few apoptotic events. These differences between heating processes may have an impact on apoptosis and do not induce detectable apoptosis with FUS alone. Whether this effect is responsible for the differences in sensitivity to RT of the various cell lines or if the anti-cancer effect of FUS could be enhanced in FaDu with more stable temperatures over time remains to be investigated.

The success of irradiation is mainly caused by damage of DNA ending in apoptotic catastrophe [24]. Generally, it is known that RT of cells prolongs the G1 and G2 phase to repair induced DNA damage [43, 52, 53]. Here, the cell population in G2/M phase increased in the RT alone and FUS+RT groups, but not in the FUS-only group, indicating an RT-induced G2 phase arrest in all cell lines. The significant recovery of FaDu and T98G cells in G2 phase probably demonstrates the occurrence of complete DNA repair with progression of the cell cycle or induction of cell death, respectively. On the contrary, no complete recovery was noticed in PC-3 cells, implying a slightly higher radiosensitivity of this prostate cancer cell line compared to the other used cell lines or a prolonged time for DNA repair in PC‑3. In the context of prolonged G1 and G2 phases, it needs to be noted that wildtype p53 protein is required for G1 phase arrest and apoptosis [54]. Since only human p53-mutant cancer cell lines were used in this study, an arrest in G1 was not expected.

The increase of direct DNA damage as DSBs was displayed as the foci number of the phosphorylated histone protein H2A.X (γH2A.X), while histone phosphorylation is the first marker of DNA DSBs [55]. Combination of FUS and RT leads to significant enhancement of initial and residual foci in T98G and PC-3 cells compared to RT alone, highlighting the radiosensitization effects of FUS. The significantly higher numbers of initial foci in T98G and PC-3 cells are probably caused by the inhibition and damage of DNA repair proteins [41]. Regarding detection of a limited recovery of foci in the RT and FUS+RT groups, we hypothesize that these DSBs are difficult to repair or remain unrepaired unless the cells die [56]. The pan-γH2A.X signal indicated the damage of S‑phase cells after combination treatment. S‑phase cells are hypersensitive to heat-induced killing, as reported by Wong [57]. The DNA DSBs of S‑phase cells occur due to the encounter of DNA replication forks with single-strand breaks [58]. When the breaks were not repaired, persistent DNA damage signal were detected. Only a few increases in foci were observed in the head and neck cell line after FUS+RT treatment compared to RT alone, illustrating that the radio-additive effects are cell type dependent. This finding in DSBs is supposed to be the reason for no reduction in the cellular metabolic activity in FaDu cells. The potential molecular mechanism may explain the different reaction of FaDu cells. Epidermal growth factor receptor (EGFR) is overexpressed in head and neck cell lines, ionizing radiation induces translocation of EGFR into the nucleus [59] and leads to activation of PI3K and RAS pathway, which supports proliferation and survival of head and neck tumor cells. Additionally, translocation of EGFR may result in increased repair of radiation-induced DSBs and consequently increase the radioresistance of head and neck cells [60]. Therefore, the results indicate that radiosensitization events induced by FUS are not a general phenomenon, a finding that is in accordance with previous reports [61, 62]. Potential mechanisms in molecular level need further investigation.

Notably, application of FUS treatment alone demonstrated no impact on cell metabolic activity, apoptosis, cell cycle distribution, and DNA damage, indicating that FUS in the applied temperature range is not a lethal treatment modality but can cause sensitization of tumor cells to RT. Chae et al. reported that HIFU-induced HT at 42–43 °C for 40 min could be tolerated by mice and showed a limited impact on tumor growth compared to the untreated control group [63].

Radiosensitizing effects of HT are still discussed controversially in the clinic. A clinical phase II study in prostate cancer patients showed a significant improvement in survival rates after combination of transrectal ultrasound HT at 42 °C and RT compared to RT alone [64]. In contrast, a randomized trial in head and neck cancer patients demonstrated no significant differences in tumor response when HT was used as adjuvant therapy for RT [65]. This is important as it reflects different sensitivities to the combination of HT and RT depending on the tumor entities.

We provide a standard FUS treatment system for in vitro study and some general observations regarding the molecular mechanism of FUS were made. However, there are several limitations in our study due to practical aspects. To measure the temperature inside the wells without perturbing the ultrasound field during the FUS treatment, an infrared thermal camera was used, as the usually applied metal thermocouples are known to disrupt the US field and lead to unnatural heating at the thermocouple surface [66]. As a compromise for temperature measurement, the ultrasound waves are reflected at the medium–air interface and the standing waves in our experimental setup were simulated (Supplementary Fig. S2). Moreover, cavitation events initiate at pressures above 0.7 MPa at 0.74 MHz, according to the report of Giesecke et al. [67]. Since the cell culture medium was not degassed, cavitation events might occur during FUS exposure and have potential biological effects on cancer cells.

Since HT has been reported in vivo and in clinical settings to sensitize tumor cells to RT by reducing tumor hypoxia and increasing tumor perfusion [21, 68], these complex processes cannot be depicted in an in vitro study; here are the limits of our preclinical study presented in this work.

## Conclusion

Our study with the in vitro FUS experimental setup demonstrated that combination of FUS-induced HT and RT resulted in a significant radiosensitization effect seen in the reduction of metabolic activity based on the enhancement of apoptosis levels and DNA double-strand breaks found in the human glioblastoma cell line T98G and prostate cancer cell line PC‑3. Our results suggest that FUS treatment alone does not harm cancer cells and has great potential to enhance the effects of RT. This is currently being further investigated in vivo.

## Supplementary Information

The characterization data of the in vitro FUS system, determination of radiation dose and FUS parameters for combination experiments, detailed data of biological experiments and statistical data are included.

## Abbreviations

BSA: Bovine serum albumin
CEM43: Cumulative equivalent minutes at 43 °C
DSB: Double-strand breaks
EGFR: Epidermal growth factor receptor
FUS: Focused ultrasound
γH2A.X: Phosphorylated histone protein H2A.X
HIFU: High-intensity focused ultrasound
HT: Hyperthermia
MRI: Magnetic resonance imaging
PI: Propidium iodide
RT: Radiation therapy
US: Ultrasound
