# Supplementary material for: Focused ultrasound radiosensitizes human cancer cells by enhancement of DNA damage
Source: Strahlenther Onkol. 2021 Apr 22;197(8):730–43. doi: 10.1007/s00066-021-01774-5 (PMC8292237; doi:10.1007/s00066-021-01774-5)
Supplement: Supplementary file 1 — The characterization data of the in vitro FUS system, determination of radiation dose and FUS parameters for combination experiments, detailed data of biological experiments and statistical data are included. [file 66_2021_1774_MOESM1_ESM.docx]

**Supplementary Material**

**Characterization of the *in vitro* FUS system**

The *in vitro* FUS system for cell cultures in well plates comprises a holder for four different interchangeable customized focused transducers. In this study two transducers with frequencies of 1.142 or 1.467 MHz and Pz 26 were characterized. Depending on the characterization and preliminary experiments, these two transducer frequencies were chosen for *in vitro* experiments since the target temperature in a range of 43 - 47 °C was reached within 2 min and was relatively stable.

The FUS field of each transducer was modeled by k-wave in MATLAB, with the transducer diameter of 35 mm and curvature of 35 mm, and then compared to experimental measurements obtained during scanning in the water tank with a 0.2 mm diameter needle hydrophone (Precision Acoustics. Dorchester. UK). The 0.2 mm needle hydrophone has a sensitivity of 55nV/Pa and a frequency response range from 1 to 35 MHz. To obtain correlated information for the acoustic field in both horizontal and vertical cross-sections simultaneously, only the horizontal cross-section was scanned in the water tank (at 0.1 mm step size) and the FUS field in the vertical cross-section was reconstructed based on the recorded values at the focal point in the horizontal plane.

Figure S1A shows MATLAB simulations of horizontal and vertical cross-sections through the focal point of the applied 1.14 MHz transducer. Experimental data are presented in Figure S1B displaying a hydrophone-scanned horizontal cross-section and reconstructed vertical cross-section based on horizontal values. In Figure S1D a slice along the X-axis (Y-fixed) in the horizontal cross-sectional plane (XY) shows an increase of the acoustic pressure to 450 kPa at the focal point for the 1.142 MHz transducer. The calculated dimension of the US focal zone at - 3 dB of attenuation for the 1.142 MHz transducer is beam diameter (BD) of 1.34 mm and a beam length (BL) of 9.70 mm (Table S1). Calculated BD and BL and the estimated from hydrophone scans showed that most of the energy in the FUS field is confined inside the well, indicating that the focus point of the transducer fits into the size of the well of a 96-well cell culture plate when the focal point is positioned at the bottom of the well. Acoustic intensities in the wells were calibrated using a hydrophone (Table S2 and S3).

The measured electrical impedance in water at 24°C was 46.4 *Ω* and 46.7 *Ω* at 34°C*.* The FUS field supplied in the wells and implicitly the range of all measurable FUS parameters that have to be considered in any *in vitro* experiment depends greatly on the transducer efficiency and its electrical impedance.

**Table S1.**  Dimensions of FUS focal zone (beam diameter, beam length, full width at half maximum (FWHM)) for the two transducers and measured electrical impedance in water.

| *Frequency (MHz)* | *Beam Diameter-calculated (mm)* | *Beam Diameter (from scan) (mm)* | *Beam Length -calculated (mm)* | *Impedance in water at 24°C (Ω)* | *Impedance in water at 34°C (Ω)* |
| --- | --- | --- | --- | --- | --- |
|  |  |  |  |  |  |
| 1.142 | 1.34 | 3.04 | 9.70 | 46.4 | 46.7 |
| 1.467 | 1.25 | 1.60 | 10.94 | 54.0 | 67.1 |


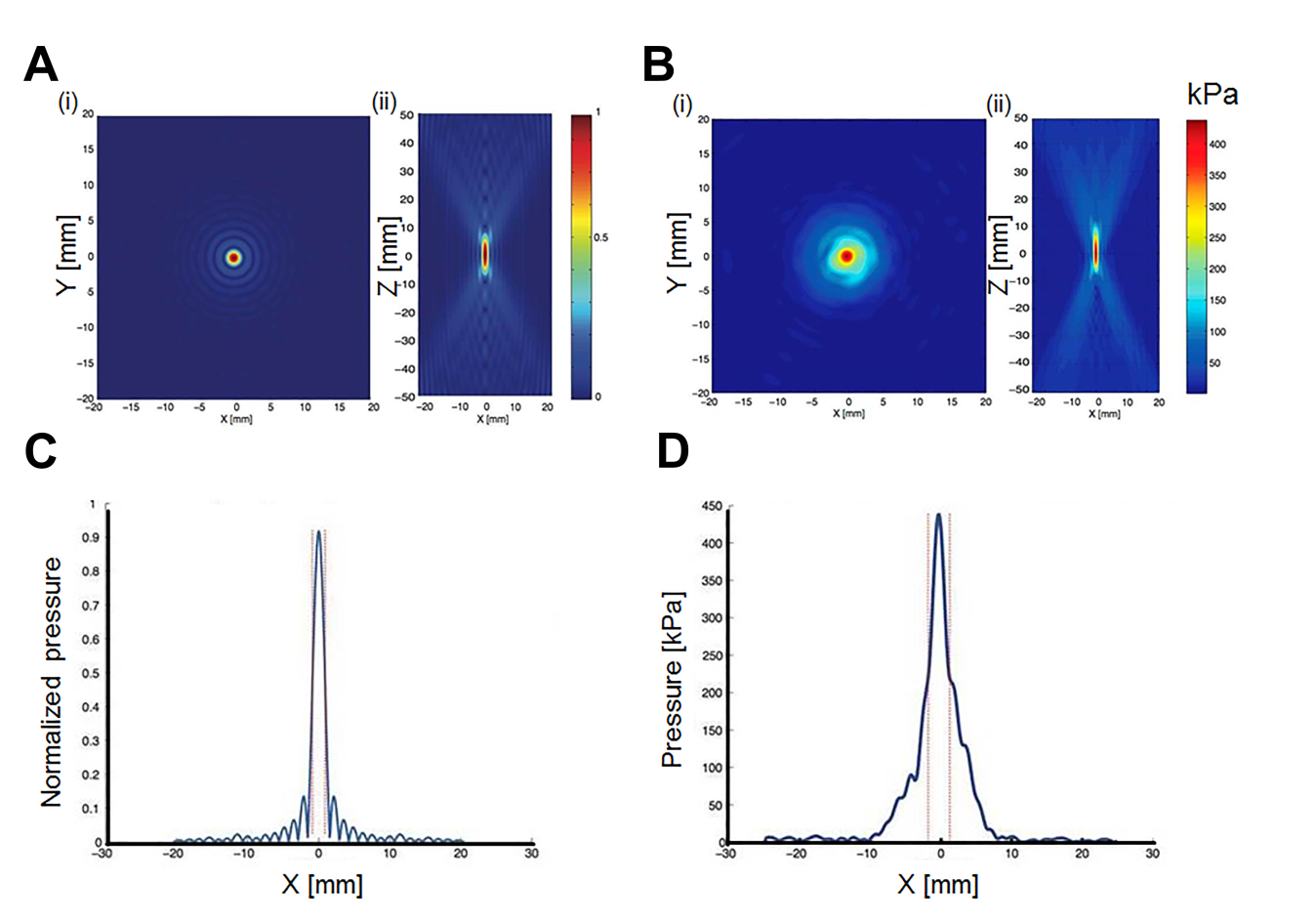


**Fig. S1. Characterization of the 1.142 MHz FUS transducer.** (A) The simulated acoustic field in horizontal cross-section through the beam at the focal point (i) and vertical cross-section at the same point (ii). The peak-to-peak pressure is represented on a normalized scale. (B) Hydrophone horizontal cross-sectional scan (i) and reconstructed field for vertical cross-section (ii) of the US field through the focal point. The peak-to-peak pressure is expressed in kPa. The beam diameter of the focal zone was estimated at 450 kPa from a slice along the x-axis: (C) from the simulated US field (beam diameter was 1.34 mm) and (D) from the hydrophone scan (beam diameter was 3.04 mm).

**Fig. S2. FUS beam profile inside the well.** Simulation of the reflections at the medium/air (left) and medium/well transition causing a standing wave inside the well. The transducer which is placed at position Z=0 mm is not visible. Due to the reflections the peak pressure decreases from 450 kPa to 270 kPa in comparison with the undisturbed acoustic field. The standing wave causes variations in the focal zone but no total annihilation can be observed.

**Table S2**. Acoustic intensity calibration of the 1.142 MHz transducer using a hydrophone

| Vpp | Acoustic intensity (W/cm2) | Acoustic pressure (MPa) | Acoustic power (W) |
| --- | --- | --- | --- |
| 0.11 | 39.05±6.56 | 0.76±0.06 | 0.55±0.09 |
| 0.19 | 87.70±5.24 | 1.14±0.03 | 1.24±0.07 |
| 0.26 | 213.01±9.59 | 1.78±0.04 | 3.00±0.14 |
| 0.35 | 306.60±18.21 | 2.13±0.06 | 4.32±0.26 |
| 0.43 | 521.84±25.68 | 2.78±0.07 | 7.36±0.36 |
| 0.5 | 835.68±28.77 | 3.52±0.06 | 11.78±0.41 |

**Table S3.** Acoustic intensity calibration of the 1.467 MHz transducer using a hydrophone

| Vpp | Acoustic intensity (W/cm2) | Acoustic pressure (MPa) | Acoustic power (W) |
| --- | --- | --- | --- |
| 0.11 | 128.80±2.91 | 1.38±0.02 | 1.58±0.04 |
| 0.15 | 224.62±8.14 | 1.82±0.03 | 2.76±0.10 |
| 0.2 | 343.89±15.20 | 2.26±0.05 | 4.22±0.19 |
| 0.25 | 538.75±18.54 | 2.82±0.05 | 6.61±0.23 |
| 0.35 | 1136.00±34.68 | 4.10±0.06 | 13.93±0.43 |
| 0.42 | 1703.86±37.82 | 5.02±0.06 | 20.90±0.46 |

**Determination of radiation dose and FUS parameters for combination experiments**

The optimal radiation dose used for further combination experiments was first determined. Human cancer cell lines were irradiated between 0 and 20 Gy and the impact of single dose irradiation on cellular metabolic activity and proliferation was measured 72 and 96 h after treatment. Loss in cell metabolic activity occurred in a dose-dependent way 72 h post-treatment with cellular activity of 98.96 ± 3.64% (FaDu), 105.05 ± 1.37% (UTSCC-8)， 69.69 ± 2.19% (T98G), 81.48 ± 5.09% (LN405), 54.05 ± 5.35% (PC-3) at 2 Gy to 72.67 ± 2.38% (FaDu) ), 88.48 ± 6.48% (UTSCC-8)， 39.34 ± 1.32% (T98G), 42.67 ± 5.05% (LN405), 16.34 ± 0.45% (PC-3) at 20 Gy. At 10 Gy, the cellular metabolic activities were significantly reduced to 89.80 ± 4.87% (FaDu), 96.06 ± 1.39% (UTSCC-8), 62.18 ± 1.37% (T98G), 83.95 ± 5.96% (LN405), and 37.49 ± 5.77% (PC-3) after 72 h. Also, proliferation was decreased to 43.66 ± 2.91% (FaDu), 44.28 ± 5.91% (UT-SCC-8), 45.57 ± 3.51% (T98G), 55.43 ± 5.75% (LN405) and 20.22 ± 0.79% (PC-3) 96 h after 10 Gy (Fig. S2). Based on calcualetd IC_50_ values in the proliferation assay of 11.29 Gy (FaDu), 7.57 Gy (UTSCC-8)，10.82 Gy (T98G), 8.49 Gy (LN405) and 3.52 Gy (PC-3) a mean single irradiation dose of 10 Gy was chosen for all FUS + RT combination experiments.


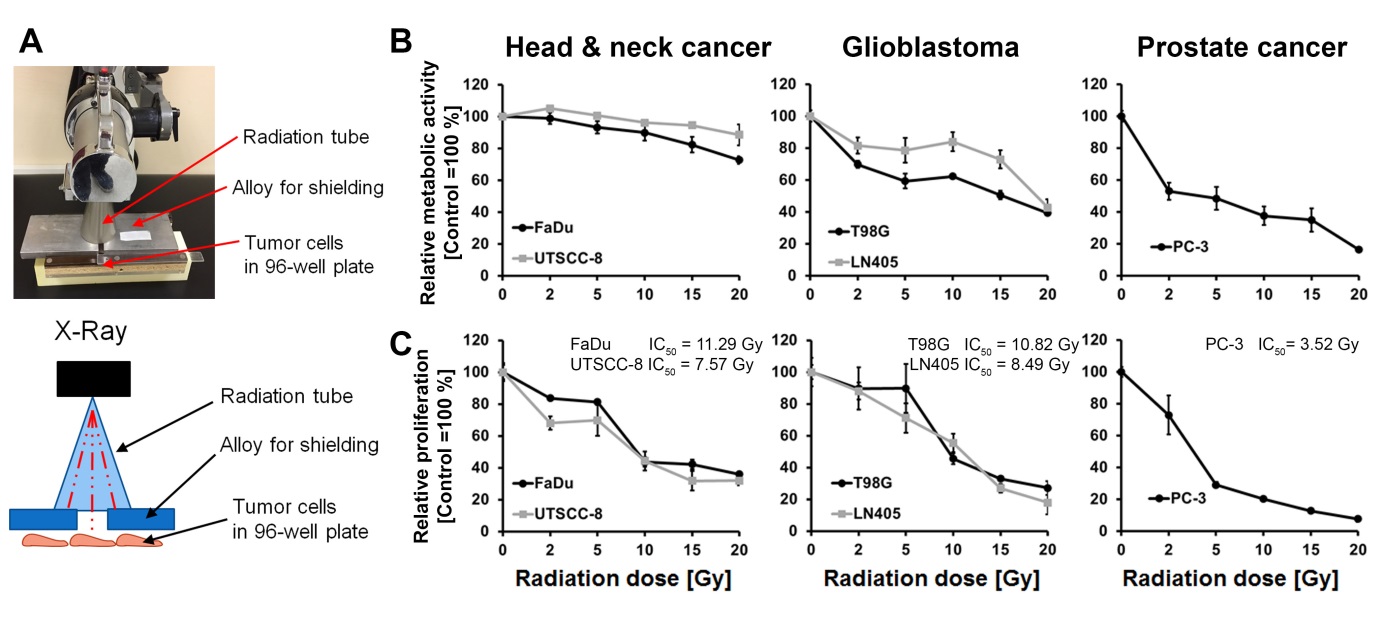


**Fig. S3: Determination of radiation dose in further combination treatments.** (A) Photography and schematic drawing of the X-ray device (DARPAC 150-MC) at the Department of Radiation Oncology, University of Leipzig, Germany. (B) Cell metabolic activity was measured with WST-1 assay 72 h after treatment. (C) Proliferation of cells was evaluated using BrdU assay 96 h after irradiation. Cell lines were cultured in 96-well plates and single irradiated at different doses. Untreated cells were set as 100%. One independent experiment in triplicates is presented as mean ± standard deviation.

**Table S4**. Cell cycle distribution after FUS and RT treatment (proportion of cells in %).

| **FaDu** | **Treatment** | **Sub-G1** | **G0/G1** | **S** | **G2/M** |
| --- | --- | --- | --- | --- | --- |
| 4 h | Control | 1.2 ± 0.99 | 50.3 ± 2.69 | 19.3 ± 1.98 | 29.65 ± 0.92 |
|  | FUS | 4.35 ± 5.44 | 40 ± 4.24 | 19.8 ± 2.69 | 36.1 ± 6.36 |
|  | RT | 4.1 ± 3.96 | 41.7 ± 0.85 * | 19.35 ± 3.61 | 33.35 ± 3.04 |
|  | FUS+RT | 6 ± 6.51 | 42.45 ± 1.48 | 19.4 ± 4.10 | 34.4 ± 0.42 * |
| 24 h | Control | 3.6 ± 3.11 | 56.25 ± 4.88 | 14.15 ± 6.15 | 25.95 ± 7.99 |
|  | FUS | 5.6 ± 4.10 | 67.65 ± 5.44 | 9.6 ± 0.99 | 17.05 ± 2.33 |
|  | RT | 5.6 ± 4.67 | 26 ± 4.88 * | 8.95 ± 2.76 | 59.35 ± 8.7 |
|  | FUS+RT | 6.1 ± 3.68 | 23.4 ± 0.71 * | 8.55 ± 2.19 | 61.95 ± 6.43 * |
| 72 h | Control | 5.2 ± 2.69 | 61.95 ± 0.07 | 9.35 ± 4.45 | 18 ± 5.94 |
|  | FUS | 4.9 ± 2.12 | 67.6 ± 4.10 | 11.75 ± 3.32 | 15.2 ± 1.98 |
|  | RT | 25.65 ±0.49 *^$^ | 31.45 ± 3.18 * | 16.45 ± 3.32 | 26.25 ± 6.29 ^$^ |
|  | FUS+RT | 32.6 ± 6.22 *^$^ | 28.15 ± 7.00 * | 10.15 ± 3.61 | 26.05 ± 0.21 ^$^ |

| **T98G** | **Treatment** | **Sub-G1** | | **G0/G1** | **S** | **G2/M** |
| --- | --- | --- | --- | --- | --- | --- |
| 4 h | Control | 6.05 ± 5.16 | 47.95 ± 11.67 | | 15.1 ± 1.13 | 30.75 ± 7.71 |
|  | FUS | 11.6 ± 12.45 | | 40.7 ± 17.96 | 14.55 ± 2.19 | 33.05 ± 7.85 |
|  | RT | 9.15 ± 4.6 | | 33.6 ± 16.97 | 18.35 ± 0.64 | 38.85 ± 13.22 |
|  | FUS+RT | 12.6 ± 13.29 | | 33.9 ± 18.53 | 18.3 ± 2.26 | 35.15 ± 7.71 |
| 24 h | Control | 6.4 ± 1.84 | | 62.05 ± 13.08 | 11.1 ± 4.53 | 15.35 ± 0.21 |
|  | FUS | 7.6 ± 3.25 | | 62.2 ± 8.63 | 12.2 ± 4.24 | 18.05 ± 1.34 |
|  | RT | 13.7 ± 0.85 * | | 13.95 ± 5.87 * | 5.35 ± 1.48 | 66.9 ± 5.23 * |
|  | FUS+RT | 24.4 ± 1.13 *^#^ | | 15.3 ± 7.64 * | 4.9 ± 0.28 | 55.35 ± 6.86 * |
| 72 h | Control | 5.6 ± 2.40 | | 53.55 ± 4.74 | 17.5 ± 2.40 | 23.4 ± 4.24 |
|  | FUS | 5.35 ± 0.35 | | 55.2 ± 1.27 | 16.9 ± 0.85 | 22.55 ± 2.05 |
|  | RT | 47.15 ± 2.90 *^$^ | | 25.55 ± 4.45 * | 12.8 ± 0.99 ^$^ | 14.45 ± 2.9 ^$^ |
|  | FUS+RT | 52.85 ± 3.75 *^$^ | | 22.55 ± 5.59 * | 11.75 ± 3.61 | 12.75 ± 5.44 ^$^ |

| **PC-3** | **Treatment** | **Sub-G1** | **G0/G1** | **S** | **G2/M** |
| --- | --- | --- | --- | --- | --- |
| 4 h | Control | 5.45 ± 4.45 | 36.15 ± 4.45 | 18 ± 2.40 | 40.35 ± 2.47 |
|  | FUS | 6 ± 3.82 | 38 ± 6.93 | 16.05 ± 0.21 | 39.8 ± 2.97 |
|  | RT | 6.75 ± 0.92 | 33.65 ± 6.58 | 18.5 ± 0.57 | 41.05 ± 8.27 |
|  | FUS+RT | 3.7 ±0.99 | 32.35 ± 10.11 | 21.3 ± 1.7 | 42.6 ± 13.01 |
| 24 h | Control | 3.8 ± 0.57 | 59.85 ± 0.35 | 11.95 ± 1.63 | 24.35 ± 1.9 |
|  | FUS | 2.4 ± 0.28 | 53.15 ± 4.03 | 11.85 ± 0.64 | 32.6 ± 4.95 |
|  | RT | 4.95 ± 1.91 | 20.3 ± 5.09 * | 12.75 ± 9.12 | 61.95 ± 16.33 |
|  | FUS+RT | 4.4 ± 1.13 | 21.55 ± 2.47 * | 11.15 ± 6.01 | 62.85 ± 7.57 * |
| 72 h | Control | 7.4 ± 3.11 | 45.8 ± 1.84 | 16 ± 0.14 | 30.7 ± 1.56 |
|  | FUS | 8.75 ± 2.75 | 45.95 ± 0.78 | 15.1 ± 3.11 | 30.1 ± 5.23 |
|  | RT | 20.65 ± 0.64 *^$^ | 16.8 ± 1.13 * | 14.4 ± 8.77 | 48.1 ± 10.47 |
|  | FUS+RT | 32.35 ± 3.32 *^#$^ | 14.8 ± 3.68 * | 9.05 ± 2.90 | 43.8 ± 2.54 * |

*, p < 0.05, significantly different from untreated control

#, p < 0.05, significantly different from RT

$, p < 0.05, significantly different from 24 h

**Table S5**. Combinatorial analysis of FUS and RT

Cell metabolic activity

| **FaDu** | | | | |
| --- | --- | --- | --- | --- |
|  | FUS | RT | FUS+RT | Theoretical sum of FUS+RT |
| 24 h | 91.446±6.554 | 94.713±7.548 | 95.278±4.739 | 88.136±11.012 |
| 48 h | 99.106±7.815 | 94.279±7.596 | 90.847±5.034 | 93.646±12.239 |
| 72 h | 99.736±2.938 | 79.789±12.198 | 87.655±7.708 | 79.426±11.509 |
| **T98G** | | | | |
|  | FUS | RT | FUS+RT | Theoretical sum of FUS+RT |
| 24 h | 94.132±8.131 | 112.677±9.714 | 100.325±6.734 | 107.420±15.094 |
| 48 h | 90.230±11.115 | 95.472±8.294 | 79.383±14.930 | 86.473±16.995 |
| 72 h | 98.933±6.359 | 58.284±14.782 | 51.856±17.128 | 57.966±15.966 |
| **PC-3** | | | | |
|  | FUS | RT | FUS+RT | Theoretical sum of FUS+RT |
| 24 h | 86.523±19.589 | 100.524±5.886 | 85.821±16.442 | 87.809±20.868 |
| 48 h | 93.607±9.123 | 72.462±8.791 | 65.759±9.721 | 67.803±10.424 |
| 72 h | 75.489±26.607 | 50.671±13.824 | 44.546±11.316 | 39.345±15.441 |

Statistic analysis

|  | P value | 24 h | 48 h | 72 h |
| --- | --- | --- | --- | --- |
| **FaDu** | RT vs. FUS+RT | 0.85401 | 0.27517 | 0.12149 |
|  | FUS+RT vs. Theoretical sum of FUS+RT | 0.82923 | 0.67274 | 0.80014 |
| **T98G** | RT vs. FUS+RT | *0.00639 | *0.01216 | 0.4066 |
|  | FUS+RT vs. Theoretical sum of FUS+RT | 0.22594 | 0.37406 | 0.44512 |
| **PC-3** | RT vs. FUS+RT | *0.02248 | 0.14453 | *0.01634 |
|  | FUS+RT vs. Theoretical sum of FUS+RT | 0.0962 | 0.53465 | 0.09369 |

DNA double-strand breaks

| **FaDu** | | | | |
| --- | --- | --- | --- | --- |
|  | FUS | RT | FUS+RT | Theoretical sum of FUS+RT |
| 1 h | 2.117±1.065 | 6.148±1.283 | 7.112±2.179 | 8.930±2.209 |
| 24 h | 1.489±0.405 | 3.608±1.349 | 5.131±0.629 | 4.676±1.441 |
| **T98G** | | | | |
|  | FUS | RT | FUS+RT | Theoretical sum of FUS+RT |
| 1 h | 10.214±2.117 | 14.282±2.930 | 20.540±6.244 | 24.466±3.158 |
| 24 h | 4.678±2.064 | 12.398±3.024 | 18.380±3.277 | 16.821±4.628 |
| **PC-3** | | | | |
|  | FUS | RT | FUS+RT | Theoretical sum of FUS+RT |
| 1 h | 2.191±0.898 | 5.999±2.608 | 10.938±0.843 | 7.405±1.473 |
| 24 h | 1.067±0.303 | 3.985±1.373 | 9.074±3.125 | 5.312±1.498 |

|  | P value | 1 h | 24 h |
| --- | --- | --- | --- |
| **FaDu** | RT vs. FUS+RT | 0.38426 | 0.20528 |
|  | FUS+RT vs. Theoretical sum of FUS+RT | 0.25604 | 0.57783 |
| **T98G** | RT vs. FUS+RT | *0.04652 | *9.80887E-4 |
|  | FUS+RT vs. Theoretical sum of FUS+RT | 0.1922 | 0.44281 |
| **PC-3** | RT vs. FUS+RT | *0.0016 | *7.80E-04 |
|  | FUS+RT vs. Theoretical sum of FUS+RT | *0.0084 | *0.0323 |
